# DNA Methylation and Hydroxymethylation in Cervical Cancer: Diagnosis, Prognosis and Treatment

**DOI:** 10.3389/fgene.2020.00347

**Published:** 2020-04-09

**Authors:** Hongming Zhu, He Zhu, Miao Tian, Dongying Wang, Jiaxing He, Tianmin Xu

**Affiliations:** Department of Obstetrics and Gynecology, The Second Hospital of Jilin University, Changchun, China

**Keywords:** DNA methylation, DNA hydroxymethylation, cervical cancer, gene, diagnosis, prognosis, treatment

## Abstract

Recent discoveries have led to the development of novel ideas and techniques that have helped elucidate the correlation between epigenetics and tumor biology. Nowadays, the field of tumor genetics has evolved to include a new type of regulation by epigenetics. An increasing number of studies have demonstrated the importance of DNA methylation and hydroxymethylation in specific genes in the progression of cervical cancer. Determining the methylation and hydroxymethylation profiles of these genes will help in the early prevention and diagnosis, monitoring recurrence, prognosis, and treatment of patients with cervical cancer. In this review, we focus on the significance of aberrant DNA methylation and hydroxymethylation in cervical cancer and the use of these epigenetic signatures in clinical settings.

## Introduction

Cervical cancer closely correlates with high rates of infection with human papillomavirus (HPV), especially HPV16 and HPV18, and is the fourth most common gynecological malignancy and second leading cause of cancer-associated female mortality globally (da Costa et al., 2019). According to new statistics, that there are 528,000 new diagnoses and 266,000 deaths annually. Notably, the global incidence of cervical cancer is higher in the urban areas as compared to that in the rural area; however, the number of patients with cervical cancer is on the rise in both areas (Meng et al., 2018). Recent studies have shown the correlation between epigenetics and development and progression of cervical cancer. Epigenetic modifications, such as DNA methylation, hydroxymethylation, demethylation, chromatin remodeling, histone modification, regulation by non-coding RNAs, and gene imprinting, are inheritable and affect genetic information without interfering with mitosis or meiosis (Xie et al., 2018). Among the epigenetic modifications, the role of candidate gene DNA methylation in cervical cancer has been studied the most. Accumulated DNA methylation in specific genes is detected as early signatures of malignant cervical cancer (Li et al., 2019a). Like methylation, DNA hydroxymethylation has gained importance recently as an epigenetic regulator of gene expression. Hydroxymethylation is involved in DNA methylation homeostasis and is mediated by the synergism between DNA methyltransferases (DNMTs) and Ten-eleven translocation (TET).

## DNA Methylation

### Mechanism of DNA Methylation

DNA methylation is a complicated process that predominantly occurs in the CpG islands of genes. DNA methyltransferases (DNMTs) transfer a methyl group to alternate cytosines, thereby generating 5-methylcytosine (5-mC). Two classes of DNMTs have been identified: DNMT1 and DNMT3 (DNMT3a and DNMT3b). DNMT1 functions in maintaining the methylation status of genes; it recognizes hemi-methylated DNA. In contrast, DNMT3a and DNMT3b are responsible for methylating genes, i.e., *de novo* DNA methylation (Wu and Zhao, 2018). DNA methylation usually results in gene silencing, whereas DNA demethylation is associated with activating gene expression.

### Aberrant DNA Methylation in Cervical Cancer

#### DNA Hypermethylation

DNA hypermethylation occurs when multiple methyl groups are transferred to one cytosine that should not be methylated, thereby resulting in gene silencing. Studies on DNA hypermethylation have provided new insights into tumorigenesis (Su et al., 2018). DNMTs are important in DNA methylation as well as hypermethylation. DNMT3b activity is one of the main factors for DNA hypermethylation (Sandhu et al., 2015). DNMT3a has been reported to be downregulated in some cancers, but DNMT1 is not known to be involved in the deregulated expression of genes (Sen et al., 2017). Since the enzyme responsible for DNA hypermethylation has been elucidated, research has shifted to determine the target genes being methylated. Clarke et al. (2017) demonstrated that, compared with healthy controls, *ADCYAP1*, *ASCL1*, *CADM1*, *DCC*, *ATP10*, *DBC1*, *HS3ST2*, *MOS*, *SOX1*, *MYOD1*, *SOX17*, and *TMEFF2* showed higher levels of methylation. *ASCL1*, *LHX8*, and *ST6GALNAC5* exhibited increased DNA methylation in cervical cancer; DNA hypermethylation also increases with the severity of cervical cancer (Kremer et al., 2018). Moreover, Verlaat et al. showed that DNA methylation usually occurs at the pre-tumorigenic stage and reaches the highest level after tumorigenesis induced by hrHPV. Twelve genes (*ANKRD18CP*, *C13orf18*, *EPB41L3*, *JAM3*, *SOX1*, *ZSCAN1*, *GHSR*, *SST*, *ZIC1*, *FAM19A4*, *PHACTR3*, and *PRDM14*) are potential biomarkers for diagnosing cervical cancer (Verlaat et al., 2018b). Clarke et al. also found that DNA methylation occurs during the transition from HPV infection to the pre-cancer stage for all the 12 carcinogenic HPV types (Clarke et al., 2018). Promoter DNA methylation regulates miRNA expression that is an important mechanism employed during the development of cervical cancer. miR-424 has been shown to be hypermethylated in its promoter and is linked to the progression of cervical cancer (Varghese et al., 2018).

#### DNA Hypomethylation

DNA methylation is performed by a complex system consisting of DNMT, DNA repair system, epigenetic regulatory factors, and environmental factors, etc. Abnormal DNA methylation (i.e., hypomethylation) affects replication and cell cycle in cells, thereby resulting in the development of tumors. During the progression of cervical cancer, rRNA levels increase. Moreover, epigenetic changes are also associated with rDNA promoter decondensation and hypomethylation. DNA methylation inhibits transcription by prohibiting the proteins from binding to methylated DNA. This can be observed in other cancers, such as lung or ovarian cancer. rRNA levels negatively correlate with the extent of methylation at the rDNA promoter; thus, rDNA hypomethylation influences cervical cancer development (Zhou et al., 2016). However, Poomipark et al. (2016) proposed a contrasting hypothesis: methyl donor status regulates DNA methylation and affects the incidence of cervical cancer. Human cervical cancer cells (C4-II) were incubated in the medium and after significant depletion of the methyl donor, DNMT3a and DNMT3b were reported to be downregulated, thereby leading to DNA hypomethylation. Thus, the status of methylation in the donor results in DNA hypomethylation; the effects of DNA hypomethylation on DNMT expression are reversible, suggesting a new idea that intake of methyl donor mediates gene expression and influences cervical cancer progression (Poomipark et al., 2016). Yin et al. (2016) have demonstrated that *STK31* inhibits apoptosis by improving cell migration and invasiveness, thereby enabling cancer progression. *STK31* is involved in hypermethylation and gene silencing. HPV16 E7 and E6/E7 oncoproteins epigenetically induce the expression of *STK31* that causes DNA hypomethylation. Thus, *STK31* is a potential candidate gene that may help the development of novel clinical approaches in the diagnosis and treatment of patients with cervical cancer (Yin et al., 2016). Varghese et al. (2018) have shown that miR-200b and miR-34c are hypomethylated during cervical cancer development.

There are target genes that undergo DNA hypermethylation and hypomethylation and can serve as cervical cancer biomarkers. However, the relevant pathways involved and other aspects of their biology remain to be understood more information still need to be studied in depth. These potential biomarkers show in Table 1.

**TABLE 1:** Potential biomarkers of DNA methylation in cervical cancer.

**Gene**	**Methylation status**	**Methylation position**	**Function**	**References**
*CADM1*	Hypermethylation	Promoter	May be related to Rb tumor suppressor pathway signaling	Mersakova et al., 2018; Del Pino et al., 2019; Rong et al., 2019
*FAM19A4*	Hypermethylation	Promoter	Not clear, maybe related to obtaining the immortal phenotype for HPV16E6E7-transduced cells	Leeman et al., 2018; Vink et al., 2019
*DcR1*	Hypermethylation	Promoter	p53-regulated DNA damage-inducible gene	Vaitkiene et al., 2013; Mansour et al., 2015; Truong et al., 2018
*PAX1*	Hypermethylation	Promoter	Tumor suppressor gene (the pathway is not clear)	Kan et al., 2014; Lai et al., 2014; Kong et al., 2015; Nikolaidis et al., 2015; Xu et al., 2015, 2018; Chen et al., 2016; Liou et al., 2016; Huang et al., 2017; Fang et al., 2019
*SOX1*	Hypermethylation	Promoter	Wnt/β-catenin signaling pathway (*SOX1* gene suppresses tumor by interacting with β-catenin)	Guan et al., 2014; Lai et al., 2014; Chen et al., 2016; Huang et al., 2017; Tian et al., 2017; Rogeri et al., 2018
*LMX1A*	Hypermethylation	Promoter	Metastasis suppressor or tumor suppressor (the regulation of *LMX1A* in carcinogenesis is not clear)	Lin et al., 2013; Rogeri et al., 2018; Xu et al., 2018
*DAPK1*	Hypermethylation	Promoter	Induce apoptosis and autophagy	Sun et al., 2015; Kim et al., 2018; Wang et al., 2018
*Rab39a*	Hypermethylation	Promoter	Regulate AKT signaling	Zou et al., 2019
*SOX11*	Hypermethylation	Promoter	Related to HPV E6 gene and TP53	Li et al., 2019c
*STK31*	Hypomethylation	Promoter/exon 1	Induced or influenced by HPV16 E7 and E6/E7 oncoproteins (epigenetic mechanism is not clear)	Yin et al., 2016
*RAPGEF1*	Hypomethylation	Promoter	Play an oncogenic role through derangement of the CRK-Rap1 signaling pathway	Samuelsson et al., 2011
*CAGE*	Hypomethylation	Promoter	Promote cell cycle progression, stimulate angiogenesis and confer resistance to anti-cancer drugs in cancer cells	Lee et al., 2006; Yeon et al., 2018

### Clinical Application of DNA Methylation in Cervical Cancer

#### DNA Methylation in the Early Diagnosis of Cervical Cancer

The development of the HPV vaccine has greatly facilitated patients; however, the incidence of cervical cancer still remains high and primarily affects younger individuals. Therefore, early diagnosis and prevention of cervical cancer is imperative and can reduce the rate of mortality. Each HPV infection has a distinct gene signature, thus, different biomarkers may be of clinical significance for the different HPV types. It is important that the sensitivity, specificity, and rate of methylation of all the biomarkers are similar (Table 2).

**TABLE 2:** Sensitivity and specificity or Methylation positivity rate of methylation diagnosis methods.

**Diagnosis method**	**HPV status**	**Function**	**Sensitivity and specificity/Methylation positivity rate**	**References**
*DAPK1* methylation detection	HPV-positive and negative	Diagnose cervical cancer	Sensitivity: 59% Specificity: 97%	Wang et al., 2018
*DAPK1* and *MGMT* methylation detection	HPV-positive and negative	Diagnose cervical cancer	Sensitivity: 43.4% Specificity: 68.6%	Sun et al., 2015
*CADM1* promoter Methylation and plasma D-dimer levels	HPV-positive	Metastasis prediction	Sensitivity: 80.4% Specificity: 90.5%	Rong et al., 2019
*CADM1*, *MAL* and *miR124* methylation detection with positive hrHPV test	HPV-positive	Diagnose HSIL/CIN2-3 and cervical cancer	Sensitivity: 80.7% Specificity: 85.1%	Del Pino et al., 2019
*FAM19A4/miR124-2* methylation detection	HPV-positive and negative	Diagnose high-risk HPV case	Methylation positivity rate of hrHPV-positive samples: 98.3% Methylation positivity rate of hrHPV-negative samples: 90.0%	Vink et al., 2019
*PAX1* methylation detection	HPV-positive and negative	Diagnose CIN3^+^	Sensitivity: 77% Specificity: 92%	Nikolaidis et al., 2015
*PAX1* methylation detection with HPV16/18 test	HPV-positive	Diagnose CIN3^+^	Sensitivity: 89.2% Specificity: 76.0%	Liou et al., 2016
*PAX1* methylation detection with Pap smearing test	HPV-positive and negative	Diagnose CIN3^+^	Sensitivity: 93% Specificity: 84%	Lai et al., 2014
*ZNF582* methylation detection with HPV16/18 test	HPV-positive	Diagnose CIN3^+^	Sensitivity: 85.4% Specificity: 80.1%	Liou et al., 2016
*SOX1* methylation detection with Pap smearing test	HPV-positive and negative	Diagnose CIN3^+^	Sensitivity: 96% Specificity: 71%	Lai et al., 2014
*LMX1A* methylation detection	HPV-positive and negative	Diagnose CIN3^+^	Sensitivity: 77% Specificity: 88%	Lai et al., 2010
*SIM1* methylation detection in plasma ccfDNA	HPV-positive and negative	Diagnose cervical cancer	Sensitivity: 38.5% Specificity: 100%	Kim et al., 2018
*SIM1* methylation detection in cervical brush specimens	HPV-positive and negative	Diagnose CIN3^+^	Methylation positivity rate: 85%	Kim et al., 2018
*ASCL1, LHX8 and ST6GALNAC5* methylation detection in lavage self-samples	HPV-positive	Diagnose CIN3^+^	Sensitivity: 74% Specificity: 79%	Verlaat et al., 2018a
*ASCL1, LHX8 and ST6GALNAC5* methylation detection in brush self-samples	HPV-positive	Diagnose CIN3^+^	Sensitivity: 88% Specificity: 81%	Verlaat et al., 2018a
*FAM19A4*, *GHSR*, *PHACTR3*, *PRDM14*, *SST* and *ZIC1* methylation detection in urine samples	HPV-positive and negative	Diagnose cervical cancer	Methylation positivity rate: 97%	Snoek et al., 2019a

##### DNA methylation biomarkers for diagnosing HPV-positive cervical cancer

Mersakova et al. (2018) and Rong et al. (2019) have recently shown that *CADM1* is a potential biomarker for cervical cancer. There was a significant difference in the promoter methylation of plasma *CADM1* and its D-dimer between healthy individuals and those with cervical cancer. Combining these factors to predict metastasis revealed high specificity (90.5%) and sensitivity (80.4%) (Rong et al., 2019). Mersakova et al. (2018) speculated that *CADM1* hypermethylation leads to suppressed Rb tumor suppressor signaling, but the exact mechanism remains to be understood. Combining the methylation of *CADM1*, *MAL*, and *miR124* with a positive test for hrHPV increases the specificity and sensitivity for detecting HSIL/CIN2-3 and cervical cancer. Methylation of *CADM1*, *MAL*, and *miR124* may be useful in estimating the risk of transformation. However, this requires further experiments to be proven conclusively (Del Pino et al., 2019).

Human papillomavirus infection, especially by HPV16 and HPV18, is a well-known cause for cervical cancer. However not all patients infected with HPV16 and/or HPV18 develop cervical cancer. Thus, screening for patients requiring therapy is problematic. High-risk HPV-infected specimens exhibit a high frequency of hypermethylation in the promoter of *DcR1* (50.0%), whereas low-risk HPV-infected samples and non-HPV-infected samples contained 16.0 and 14.6% *DcR1* hypermethylation, respectively. High-risk HPV infection silences *DcR1* expression, thereby promoting the development of cervical cancer (Truong et al., 2018). Although the pathway or mechanism employed during cervical cancer are yet to be elucidated, the mechanism employed by *DcR1* in other cancers has been reported. Vaitkiene et al. (2013) showed that *DcR1* encodes a receptor incapable of inducing apoptosis; however, it protects glioblastoma multiforme (GBM) cells from TRAIL-induced apoptosis. *DcR1* is hypermethylated and produces low levels of protein during the onset of cancer. This can explain specificity and sensitivity of cancer cells to the apoptosis-inducing activity of TRAIL (Vaitkiene et al., 2013). However, another study has identified the dependence of *DcR1* on NF-κB/p50 and shown that *DcR1* may be related to *p53* and *Bcl3* in GBM (Mansour et al., 2015). Taken together, *DcR1* may be a promising biomarker for the early diagnosis of cervical cancer and help select patients requiring early treatment.

Kremer et al. (2018) have identified three related genes, including *ASCL1*, *LHX8*, and *ST6GALNAC5*. *ASCL1* and *LHX8* correlated well with CIN3^+^ (AUC 0.79 and 0.81, respectively) and *ST6GALNAC5* correlated moderately with CIN3^+^ (AUC 0.71) of *ASCL1, LHX8*, and *ST6GALNAC5* exhibited 72.1, 73.8, and 55.7% sensitivity with CIN3^+^, respectively. In conclusion, *ASCL1* and *LHX8* have better performance and higher sensitivity, thereby making them suitable biomarkers for early diagnosis (Kremer et al., 2018). Interestingly, *ASCL1* functions as an oncogenic transcription factor in lung cancer (Lenhart et al., 2015). Other reports have not been successful in correlating *LHX8* to cancer. However, Kremer et al. (2018) have reported increasing levels of hypermethylated *ASCL1* and *LHX8* with increasing severity of cervical cancer. This could be attributed to contamination by other factors like the human immunodeficiency virus (owing to the infected patient cohort). However, Kremer et al. (2018) also showed that using one of these three genes (*ASCL1*, *LHX8*, and *ST6GALNAC5*) was not sensitive. Verlaat et al. (2018a) have described a highly effective 3-gene methylation classifier (*ASCL1*, *LHX8*, and *ST6GALNAC5*) to detect the pre- and tumorigenic stage of cervical cancer. Combining these genes improves detection sensitivity and specificity. They detected methylation in hrHPV-positive lavage and brush self-samples; thus, the 3-gene methylation classifier (*ASCL1*, *LHX8*, and *ST6GALNAC5*) exhibited excellent clinical performance in the detection of CIN3. The sensitivity and specificity in hrHPV-positive lavage and brush self-samples were 74, 79, 88, and 81%, respectively (Verlaat et al., 2018a). Thus, this can be used as a prospective method for diagnosis.

In addition to putative biomarkers for the early diagnosis of cervical cancer lesions, there is an urgent need for the identification of biomarkers for CIN2/CIN3 hrHPV-positive patients to help determine the course of treatment. Some CIN2 and CIN3 lesions develop or regress spontaneously and do not need to be treated. Due to the lack of known biomarkers, these patients receive excessive treatment. Snoek et al. (2019b) showed that three genes (*GHSR*, *SST*, and *ZIC1*) are important for such patients. These genes were significantly methylated in tissue specimens and cervical scrapes with increasing severity of the disease. Especially in advanced CIN2/CIN3 and squamous cell carcinoma (SCC), these genes are highly methylated (Snoek et al., 2019b). Thus, methylated *GHSR*, *SST*, and *ZIC1* may serve as prognostic markers for hrHPV-positive women.

##### Diagnostic DNA methylation biomarkers for HPV-positive and negative cervical tumors

Hypermethylated *PAX1* is important in cancer progression (Huang et al., 2017) that functions in regulating cellular differentiation and proliferation (Fang et al., 2019). *PAX1* can be used to detect cervical squamous cell carcinoma (CSCC) in 121 patients from eastern China with 80.9, 83.7, and 79.0% accuracy, specificity, and sensitivity, respectively (Xu et al., 2018). Combining methylated *PAX1* and the presence of HPV16/18 results in an 89.2 and 76.0% sensitivity and specificity, respectively in detecting CIN3^+^ (Liou et al., 2016). Using the methylation status of *PAX1* to determine the cancerous nature (and CIN1–3) of samples from Shanghai, China is associated with a sensitivity and specificity of 81 and 93%, respectively; however, the sensitivity and specificity for cancer versus CIN2/3 is 32% and 90%, respectively (Xu et al., 2015). Single mutations in *PAX1* have been accurate in diagnosing cervical/HSIL in a group of 15 individuals with a specificity and sensitivity of 89% and 80%, respectively (Kong et al., 2015). Lai et al. (2014) have demonstrated that methylated *PAX1* can be used for detecting CIN3^+^ lesions with a sensitivity and specificity of 64% and 91%, respectively in Taiwanese patients. Interestingly, combining results from Pap smears with parallel testing of *PAX1* results in superior specificity (84%) and similar sensitivity (93%). Using a combination of Pap smear results with HPV testing leads to an increase in specificity and sensitivity of 66 and 97%, respectively. Thus, using Pap smears and *PAX1* parallel testing results in better diagnosis (Lai et al., 2014). Kan et al. (2014) have also described the importance of combining *PAX1* testing with Pap smears. Combining *PAX1* with Pap testing, the sensitivity and specificity were 89 and 83%, respectively (Kan et al., 2014). A meta-analysis comprising Asian individuals revealed that the sensitivity and specificity of *PAX1* methylation is 73 and 87%, respectively in HSIL/CIN3^+^/cervical cancer patients (Chen et al., 2016). Another meta-analysis used 1,385 individuals with different stages of CIN to show that the sensitivity and specificity of *PAX1* methylation in CIN3^+^ samples are 77% and 92%, respectively (Nikolaidis et al., 2015). In summary, different geographical locations and various methods (especially using combinations) affect the sensitivity and specificity of detecting *PAX1* methylation. Thus, *PAX1* methylation can help identify CIN3^+^ patients in clinical settings.

*LMX1A* has been recently reported to be hypermethylated in cervical cancers (Rogeri et al., 2018). *LMX1A* is a suppressor of tumorigenesis and metastasis of cervical cancer; however, the regulation of *LMX1A* during tumorigenesis remains to be understood. Lin et al. (2013) have provided two hypotheses for the mechanism of action employed by *LMX1A*. In conditions of high promoter methylation, Sp1 binds to the promoter of *LMX1A* and inhibits *LMX1A* expression. Second, overexpression of *EZH2* suppresses *LMX1A* expression in cancer cells. Thus, *Sp1* and *EZH2* may work in concert to regulate *LMX1A* expression (Lin et al., 2013). The sensitivity, specificity, and accuracy of the detection of cervical cancer are 63.3, 35.7, and 89.3%, respectively using *LMX1A* (Xu et al., 2018). However, another group have used *LMX1A* methylation to detect CIN3^+^ in samples with moderate sensitivity (77%) and specificity (88%) (Lai et al., 2010). Thus, whether *LMX1A* can be used as a biomarker remains to be confirmed.

Upon analyzing various sets of data and cross-referencing, *CDH1*, *CDKN2A*, *RB1*, and *TP53* are putative biomarkers; these genes need to be studied for their role in diagnosing cervical cancer (Cardoso et al., 2017). Kim et al. (2018) have determined the methylation status of *SIM1* in circulating cell-free DNA and cervical brush specimens by quantitative methylation-specific polymerase chain reaction, indicating that hypermethylated *SIM1* is a potential biomarker for cervical cancer. Detecting methylated *SIM1* in diagnosing cervical cancer was 38.5 and 100% sensitive and specific, respectively. Since the data is not too promising, it needs improvement. For cervical brush specimens, positive methylated *SIM1* was detected in 0, 0, 5.3, 41.2, and 85% of normal, CIN1, CIN2, CIN3, and cancer samples, respectively (Kim et al., 2018). The proportion of methylated *SIM1* in cells increases at the CIN3 stage; *SIM1* hypermethylation mediates the transition from CIN3 to invasive cervical cancer. Therefore, detecting *SIM1* methylation at the CIN3 stage can help prevent the progression of cervical cancer.

Numerous studies have reported the potential of *CADM1*, *MAL*, *miR-124*, *SOX1*, *ERT*, and *EPB41l3* as biomarkers. *DCC* was identified to be a crucial candidate as a methylation biomarker during the screening of cervical cancer. *DCC* is involved in cell cycle control and apoptosis and can be found in several cancers, such as ovarian cancer, etc. However, experiments in the future need to confirm if methylated *DCC* can serve as a marker during the screening of cervical cancer (Clarke et al., 2017).

Sun et al. (2015) analyzed the methylated promoters of two genes (e.g., *DAPK1* and *MGMT*, *MGMT* and *RARB*, and *DAPK1* and *RARB*) using methylation sensitive-high resolution melting analysis to determine if this method enhances the sensitivity and specificity of clinical diagnosis. Methylation of *DAPK1* combined with *MGMT* has a sensitivity and specificity of 43.4 and 68.6%, respectively; this may help develop novel methods to increase the rate of successful diagnosis (Sun et al., 2015).

Vink et al. (2019) reported that combining *FAM19A4/miR124-2* methylation with hrHPV testing was more efficient. They assessed cervical cancer globally to find more than 98% cases of *FAM19A4/miR124-2* methylation. *FAM19A4/miR124-2* methylation can be detected in hrHPV-negative carcinomas; thus, combining these methods complements each other and reduces the rate of missed diagnoses (Leeman et al., 2018; Vink et al., 2019).

Various self-detection methods for cervical cancer have become increasingly popular over the years owing to their convenience of use. Van Ostade et al. (2018) proposed the use of cervical vaginal fluid in screening cervical cancer by self-detection. α-actinin-4 might play a significant role in the detection of cervical cancer from cervical vaginal fluid. Although this method requires significant improvement, it provides an exciting prospect for the early diagnosis of cervical cancer (Van Ostade et al., 2018). Snoek et al. (2019a) have demonstrated that collecting urine is more convenient and comfortable for patients than cervical scrapes and is a promising alternative testing material to screen for cervical cancer. hrHPV testing was conducted from urine sediments, native urine, and cervical scrapes, concluding a near-perfect agreement. The methylation levels of six genes (*FAM19A4*, *GHSR*, *PHACTR3*, *PRDM14*, *SST*, and *ZIC1*) showed differential expression in the urine sediments. The rate of methylation for each marker was at least 97% (Snoek et al., 2019a). Thus, urine-based DNA methylation testing is a novel and promising method that could be used for the diagnosis of cervical cancer.

#### DNA Methylation in Prognosis and Treatment

The biomarkers useful for the prognosis of cervical cancer remain to be elucidated. The treatment of cervical cancer is limited to chemoradiation therapy, surgery, and drug therapy. However, more targets need to be identified for use in the prognosis and treatment of cervical cancer. Table 3 lists the potential for methylation-based therapy against cervical cancer.

**TABLE 3:** The summary of the potential methylation therapy.

**Method**	**Related gene**	**Function**	**Advantages and disadvantages**	**References**
Combine chemoradiation therapy with *DAPK1* gene target	*DAPK1*	Suppress the proliferation of tumor cells and induce apoptosis and autophagy	Advantages: Decrease side effects Disadvantages: Not mentioned	Bhattacharjee et al., 2018; Huang et al., 2018; Ren et al., 2018; Sood et al., 2018; Wang et al., 2018; Yadav et al., 2018
Combine chemoradiation therapy with *BRCA1* gene target	*BRCA1*	DNA damage repair, transcriptional regulation and apoptosis	Advantages: Decrease side effects Disadvantages: The overexpression of BRCA1 gene may improve tumorigenesis	Li et al., 2018a; Maresca et al., 2018; Sood et al., 2018; Sun et al., 2018
Combine chemoradiation therapy with *MGMT* gene target	*MGMT*	Repair cytotoxic lesions by removing the methyl adducts from DNA	Advantages: Decrease side effects Disadvantages: Not mentioned	Jiapaer et al., 2018; Ren et al., 2018; Scicchitano et al., 2018; Sood et al., 2018; Yuan et al., 2018
Menthol	*FANCF*	Reverse the hypermethylation of *FANCF* promoter	Advantages: Menthol has high solubility and bioavailability Disadvantages: Not mentioned	Parashar et al., 2017
EGCG with eugenol-amarogentin	*LimD1* and *P16*	Suppress proliferation and induce apoptosis through promoting the hypomethylation of *LimD1* and *P16* promoters	Advantages: More effective Disadvantages: Not mentioned	Pal et al., 2018
Combine chemoradiation therapy with *STK31* gene target	*STK31*	Suppress migration and invasiveness and induce apoptosis	Advantages: Increase the efficacy of chemoradiation and reduce chemoresistance Disadvantages: Epigenetics drugs have many restrictions	Yin et al., 2016
Quercetin	*TSG*	Downregulate the levels of global DNA methylation and reverse the hypermethylation of *TSG* promoter	Advantages: Natural and fewer side effects Disadvantages: Low bioavailability	Kedhari Sundaram et al., 2019
Limoniastrum guyonianum aqueous gall extract (G extract) and luteolin	*p16INK4A*	Suppress the proliferation and induce apoptosis by activating *p16INK4A*-dependent cell cycle checkpoint signaling pathway	Advantages: Natural and fewer side effects Disadvantages: Not experiment *in vivo* and have no idea if it works for patients	Krifa et al., 2013

Cisplatin-based neoadjuvant chemotherapy (NACT) is performed before surgery depending on the treatment of large or locally advanced squamous cervical cancer and followed by radical hysterectomy. This novel treatment strategy minimizes tumor volume, controls micrometastasis, and enhances the integrity and safety of surgery. However, the resistance and efficacy of NACT before chemotherapy needs to be assessed. Zhu et al. (2017) used immunohistochemistry and bioinformatic analyses to identify IB2 or IIA2 cervical cancer patients; the expression of galectin-1 and integrin α5β1 correlates with cisplatin-based NACT. Among patients with cervical squamous cell carcinoma, the expression of galectin-1 and integrin α5β1 in tumor cells and stromal cells is downregulated after NACT. The expression of galectin-1 and integrin α5β1 is significantly higher in patients with poor NACT efficacy than that of NACT; thus, the downregulation of galectin-1 and integrin α5β1 after chemotherapy is not significant among the patients with poor NACT efficacy. The resistance and prognosis of NACT can be determined by the expression of galectin-1 and integrin α5β1 followed by the selection of a suitable treatment plan for patients (Zhu et al., 2017).

Although chemoradiation therapy is an efficient mode of treatment, it is associated with side effects that result in inefficient prognosis of cervical cancer. Thus, closely related genes were tested to determine if chemoradiation therapy induces epigenetic modifications and regulates gene expression in cervical cancer (Sood et al., 2018). *DAPK1* methylation was observed to be decreased with a concomitant increase in the *DAPK1* transcripts in the tissue after chemoradiation therapy. *DAPK1* has been shown to be associated with the severity of cervical cancer (Wang et al., 2018); *DAPK1* upregulation suppresses tumor cell proliferation and improve apoptosis and autophagy (Bhattacharjee et al., 2018; Huang et al., 2018; Ren et al., 2018; Yadav et al., 2018). Thus, chemoradiation has the capacity to increase *DAPK1* mRNA levels and reduce the number of tumor cells. This suggests that determining the expression of *DAPK1* during chemoradiation therapy will help reduce side effects.

Interestingly, *BRCA1* is involved with the same phenotype after chemoradiation therapy (Sood et al., 2018). However, *BRCA1* functions differently as compared to *DAPK1*; *BRCA1* is a tumor suppressor (Li et al., 2018a). It predominantly regulates DNA damage repair, transcription regulation, and apoptosis (Maresca et al., 2018; Sun et al., 2018). Therefore, it can serve as novel target during chemoradiation, like *DAPK1*. Overexpression of *BRCA1* is involved in the development of cancer (Li et al., 2018a).

*MGMT* is another candidate that shows decreased methylation (Ren et al., 2018). *MGMT* repairs cytotoxic lesions by removing methyl adducts from DNA (Jiapaer et al., 2018; Scicchitano et al., 2018; Yuan et al., 2018), indicating *MGMT* can be a therapeutic target. Sood et al. (2018) have also tested the potential of *ESR1*, *MYOD1*, and *MLH1*. ESR1 transcript levels increase in the tissue, but decrease for *MYOD1* and *MLH1*. However, it is unclear whether epigenetic modifications of these genes influence the development of cervical cancer after chemoradiation therapy. Moreover, their functions and associated pathways in tumor cells need to be understood in sufficient details.

Hypermethylated promoter of *FANCF* can be frequently detected in patients with cervical cancer. However, epigallocatechin-3-gallate (EGCG) and -(-)Menthol reverse the hypermethylation in the *FANCF* promoter. Upon inhibiting M. SssI (DNMT1 analog), these two substances inhibited DNMT1, thereby hypomethylating and reactivating the *FANCF* promoter and inhibiting cervical cancer progression (Parashar et al., 2017). A similar study reported that as compared to individual treatment, EGCG and eugenol-amarogentin strongly inhibits cellular colony formation and proliferation and induces apoptosis via promoting the hypomethylation of the promoters of *LimD1* and *P16*. Thus, this combination of EGCG and eugenol-amarogentin may be a more efficacious line of therapy (Pal et al., 2018).

*STK31* is a new potential therapeutic target in cervical cancer, especially in HPV16/18-positive cervical tumors. *STK31* expression can be induced by the HPV16 E7 and E6/E7 oncoproteins by regulating the methylation status of *STK31* in the HPV16/18-positive cells, while HPV-negative cervical cancer cells exhibit silencing of *STK31*. Therefore, inhibiting the tumorigenic activities of *STK31* in HPV16/18-positive patients can be used in treating cervical cancer, indicating that *STK31* is a crucial target. Moreover, epigenetic drugs increase the efficacy of chemoradiation and reduce chemoresistance. However, not all patients can be administered epigenetics drugs owing to the many restrictions for use in clinical application (Yin et al., 2016).

*CDKN2A* can also prevent the progression of cervical cancer since patients frequently have hypermethylated *CDKN2A*. The absence of *p16INK4A* protein inhibits the expression of HPV E7 oncoprotein and cervical cancer progression. *CDKN2A* methylation positively correlates with *p16INK4A/p14ARF* expression in head and neck cancer. There may be a similar correlation in cervical cancer. Therefore, the decrease in methylation of *CDKN2A* may lead to reduced protein levels of *p16INK4A* and HPV E7 oncoprotein, thereby inhibiting cervical cancer progression. Thus, *CDKN2A* is an important therapeutic target (Wijetunga et al., 2016).

Researchers put more focus on using chemical medicine and radiochemotherapy for the treatment of cancers; the function of natural substances, like vegetables or fruits, is usually overlooked. Recently, Kedhari Sundaram et al. (2019) demonstrated that quercetin, a phytochemical from vegetables and fruits, possesses anti-cancer activity by downregulating global DNA methylation and reversing hypermethylation in the *TSG* promoter, thereby activating *TSG* expression. However, for clinical application, the bioavailability of quercetin is influenced by factors including source, gender, and form of quercetin. Targeted delivery and liposomal and nanoparticle-based delivery to tumors are methods that improve the bioavailability of compounds and are currently under improvement. Thus, in the future, quercetin is expected to be a safer therapeutic for cervical cancer. Krifa et al. (2013) have demonstrated the anti-cancer effect of the aqueous gall extract (G extract) of Limoniastrum guyonianum and luteolin. These two natural products downregulate UHRF1 and DNMT1 via global DNA hypomethylation and activate *p16INK4A*-dependent cell cycle checkpoint signaling. Thus, G extract and luteolin inhibit cervical cancer cell proliferation and induce programmed cell death (Krifa et al., 2013). However, they have only used HeLa cells *in vitro*. The efficacy and association with side effects still remain to be understood.

## DNA Hydroxymethylation

### Mechanism of DNA Hydroxymethylation

**FIGURE 1:**
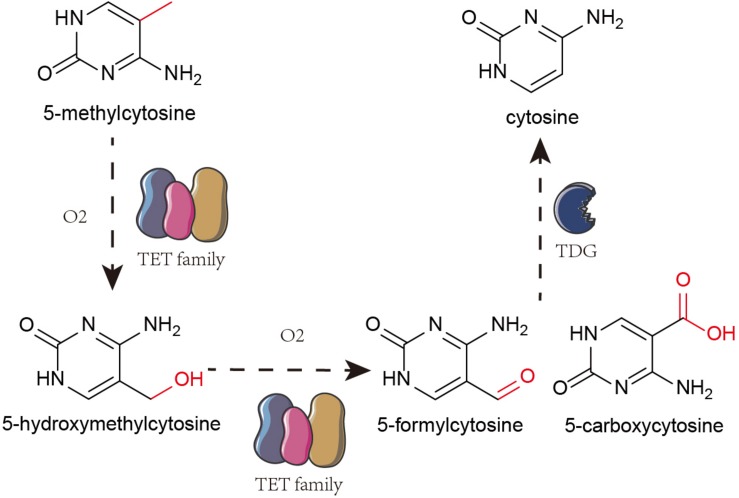
Mechanism of DNA hydroxymethylation. TET family oxidize 5-mc to 5-hmC. And then, TET proteins further oxidize 5-hydroxymethylcytosine (5-hmC) into 5-formylcytosine and 5-carboxylcytosine. Finally, it removes the carboxyl group from 5-formylcytosine and 5-carboxylcytosine by TDG to restore them to unmethylated cytosine.

The Ten-eleven translocation (TET) family oxidize 5-mC to 5-hydroxymethylcytosine (5-hmC) that is further oxidized to 5-formylcytosine and 5-carboxylcytosine. Finally, using thymine-DNA glycosylase (TDG), the carboxyl group from 5-formylcytosine and 5-carboxylcytosine are removed to restore unmethylated cytosine pool (Figure 1; Silva et al., 2017; Jayanthi et al., 2018; Zhu et al., 2018). 5-hmC is an intermediate throughout the process (Li et al., 2016).

### Aberrant DNA Hydroxymethylation in Cervical and Other Cancers

Compared with the tissues from a normal cervix, the expression of 5-hmC and TET2 decrease in the cervical cancer tissues while the expression of TET1 and TET3 remain unaltered. Thus, the decreased expression of TET2 may decrease 5-hmC (Zhang et al., 2016), suggesting 5-hmC to be a putative prognostic biomarker. Other novel (potential) biomarkers in cervical cancer that undergo hydroxymethylation include *ACTG1*, *SALL3*, *DNAJA3*, *SERPINB6*, *CDC14B*, and *CALN1* (Wang et al., 2019).

Wu et al. (2019) have demonstrated the downregulation of TET1 and 5-hmC in pancreatic tissues and cell lines. Pancreatic cancer patients with high TET1 levels exhibit longer overall survival than patients with low levels of TET1. Therefore, TET1 can suppress pancreatic tumor development by inhibiting proliferation and metastasis. This is achieved via Wnt signaling where TET1 binds and demethylates the promoter of the secreted frizzled-related protein 2 (*SFRP2*) to activate *SFRP2* transcription (Wu et al., 2019). Interestingly, TET1 can suppress colon cancer, wherein TET1 binds to the promoter of *DKK* and inhibits Wnt signaling to maintain the hypomethylated state (Neri et al., 2015). Thus, TET1 may be a promising therapeutic in the future.

Glioblastoma melanoma and myeloid cancers are associated with reduced 5-hydroxymethylation. In ∼9% of patients with nasopharyngeal carcinoma, there is a mutation in TET1, TET2, or TET3 (Mohr et al., 2011; Lin et al., 2014; Wille et al., 2015; Salgado et al., 2020). There is a decrease in 5-hmC in melanoma samples as compared to that in nevus samples (Salgado et al., 2020). Wille et al. (2015) have shown that the inhibition of TET protein activity promotes lytic EBV promoter methylation in normal oral keratinocyte cells, thereby increasing Z-mediated lytic reactivation and inhibiting R-mediated lytic reactivation. The *BRLF1* promoter contains a CpG-containing Z-binding site. Methylation of this region leads to Z-mediated viral reactivation. However, the presence of 5-hmC in this site in normal oral keratinocyte cells prevents Z-mediated viral reactivation. Thus, the inhibition of TET protein activity and low levels of 5-hmC function in the development of nasopharyngeal carcinoma (Wille et al., 2015).

There is a global decrease in hydroxymethylation in tumors that has elucidated the role of hydroxymethylation in tumor suppression. Li et al. (2019c) demonstrated that, although 5-hmCs were intermediates during oxidation, a proportion of 5-hmCs can be found in the genome. Hydroxymethylated *P16* alleles are transcriptionally inactive and hydroxymethylation increases reactivation of *P16* in cancer cells (Li et al., 2019b).

Genome-wide DNA hydroxymethylation has been considered a sensitive biomarker for prostate cancer (PCa) detection. Owing to the loss of hydroxymethylation, different cancers maintain low levels of 5-hmC (Feng et al., 2015; Strand et al., 2015; Kamdar et al., 2016; Spans et al., 2016). However, Grelus et al. (2017) have shown that hydroxymethylation markers cannot be single biomarkers for PCa diagnosis. They should be used as a supplement for prostate-specific antigen to diagnose PCa. Although hydroxymethylation can be detected in multiple cancer types, the utility of genomic hydroxymethylation in diagnosing cancer may only be restricted to one type of cancer (Grelus et al., 2017). Thus, whether DNA hydroxymethylation can be used in clinical diagnosis needs to be explored. Even so, we have concluded the function of DNA hydroxymethylation in various kinds of tumors as a reference in Table 4.

**TABLE 4:** The function of DNA hydroxymethylation in various kinds of tumors.

**Related TET proteins**	**Related gene**	**Cancer types**	**Function**	**References**
TET2	Not mentioned	Cervical cancer	Decreased expression of TET2 may be the mechanism for decreased 5-hmC	Zhang et al., 2016
TET1/2/3	*ACTG1*, *SALL3*, *DNAJA3*, *SERPINB6*, *CDC14B*, *CALN1*	Cervical cancer	May be related to Wnt, MAPK, Rap signaling pathways	Wang et al., 2019
TET1	*SFRP2*	Pancreatic cancer	Inhibit Wnt signaling pathway by catalyzing demethylation to activate transcription of *SFRP2*	Wu et al., 2019
TET1	*DKK*	Colon cancer	Inhibit Wnt signaling pathway by binding TET1 to the promoter of the DKK gene inhibitors to maintain them hypomethylated	Neri et al., 2015
TET1/2/3	*PTEN*	Melanoma	Activate the *PTEN* promotor	Salgado et al., 2020
TET1/2/3	*P16*	All cancer types	Hydroxymethylation increases the reactivate potential of the *P16* gene	Li et al., 2019b

## Correlation Between DNA Methylation and Hydroxymethylation in Cancer

Several studies have highlighted the importance of 5-hmC in tumorigenesis. TET proteins are responsible for the presence of mutations or downregulated gene expression in tumorigenesis, thereby reducing the 5-hmC content in malignant cells (Haffner et al., 2011). The catalytic co-substrate α-ketoglutarate may greatly influence TET protein activity. *IDH1* and *IDH2*, that produce α-ketoglutarate, can be present in mutated forms in many different kinds of cancers. Mutated *IDH1* and *IDH2* are incapable of forming α-ketoglutarate and 2-hydroxyglutarate, thereby inhibiting TET protein activity and stimulating the development of cancer (Kamdar et al., 2016).

**FIGURE 2:**
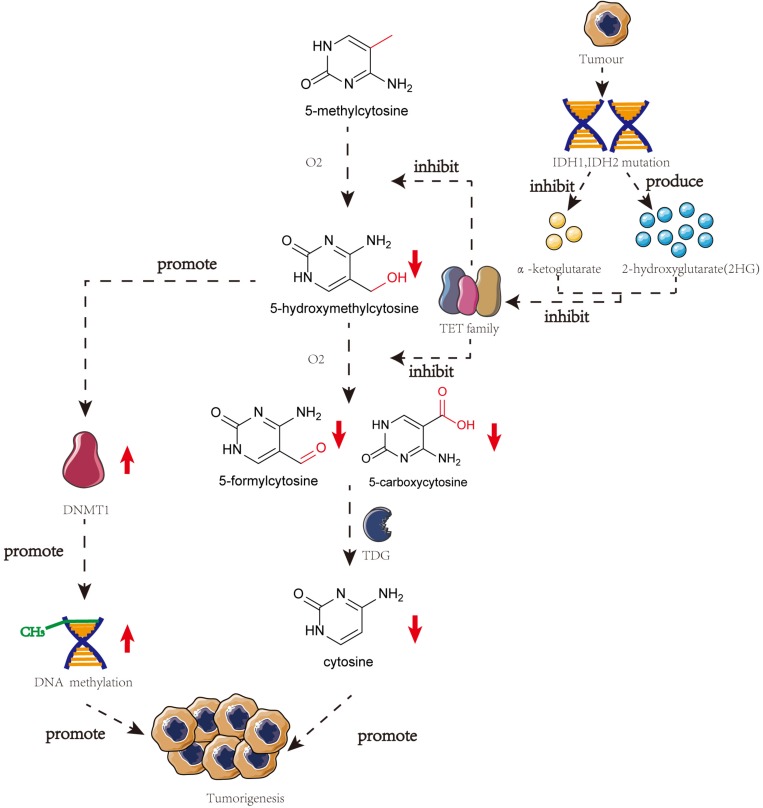
The crosstalk of DNA methylation and DNA hydroxymethylation in the progress of the tumor. In many different kinds of cancers, IDH1, and IDH2 will mutate which can restrain the production of α-ketoglutarate and produce 2HG. Both of these two phenomena will lead to the inhibition of TET family and meanwhile, the low level of 5-hmC can promote the activity of DNMT1. These two pathways will finally result in the tumorigenesis.

Genome-wide loss of 5-hmC does not correspond with the global loss of 5-mC, indicating that the altered levels of 5-hmC in tumorigenesis may involve an independent mechanism (Ficz and Gribben, 2014). However, some studies have demonstrated a correlation between DNA methylation and hydroxymethylation. When the DNA substrate contains 5-hmC, DNMT1 activity is reduced a lot (∼60-fold), thereby diluting the 5-mC pool. Low levels of 5-hmC promote DNMT1 activity (Li et al., 2018b). Using clinical samples, Wang et al. (2019) have demonstrated that 5-hmC negatively correlates with cervical cancer progression and is downregulated upon regulation of 5-mC. Thus, demethylation and hydroxymethylation are inhibited and there is a global accumulation of aberrant DNA hypermethylation in cervical cancer (Figure 2; Wang et al., 2019).

## Conclusion

The incidence and development of tumors depend on multiple factors, including internal and external factors. Researchers have studied genetic factors in the past. However, there has been an increase in the focus on epigenetics to study gene function and genetic modification in the hope that epigenetics may be the key to developing other novel methods in the early diagnosis or treatment of diseases. In this review, we have summarized the methylation-specific modifications found in cervical cancer-related genes and their clinical application, thereby highlighting the aforementioned importance of epigenetics in cervical cancer. We have also described how DNA hydroxymethylation influences tumorigenesis simultaneously with DNA methylation. These phenomena may provide new methods and strategies for the early prevention and diagnosis, monitoring recurrence, and prognosis and treatment of cervical cancer. However, owing to various restrictions, a large number of samples and detailed research is required for some methods that cannot be used for clinical application yet.

## Author Contributions

HoZ drafted the manuscript. HeZ and MT revised the manuscript. JH and DW contributed to the acquisition and interpretation of data. TX designed the work. All authors contributed to the manuscript revision, read, and approved the submitted version.

## Conflict of Interest

The authors declare that the research was conducted in the absence of any commercial or financial relationships that could be construed as a potential conflict of interest.
